# Safety population size and duration of exposure prior to approval of new medicines: A database analysis of medicines centralised approved in the European Union between 2011 and 2023

**DOI:** 10.1371/journal.pone.0342698

**Published:** 2026-02-09

**Authors:** Luísa Bouwman, Hubert Leufkens, Bruno Sepodes, Carla Torre

**Affiliations:** 1 Faculdade de Farmácia, Universidade de Lisboa, Lisboa, Portugal; 2 Laboratory of Systems Integration Pharmacology, Clinical and Regulatory Science, Research Institute for Medicines of the University of Lisbon (iMED.ULisboa), Lisboa, Portugal; 3 Division of Pharmacoepidemiology and Clinical Pharmacology, Utrecht Institute for Pharmaceutical Sciences (UIPS), Utrecht University, Utrecht, The Netherlands; The kids Research Institute Australia, AUSTRALIA

## Abstract

**Background:**

This study aims to analyse the number of patients who had been exposed to a new medicine before the approval by the European Commission (EC), as well as the number of patients studied long-term for chronic medication use. The results were compared with the International Council for Harmonisation’s (ICH) E1 guideline recommendations.

**Methods:**

All medicines containing new active substances approved between 2011 and 2023 were included in the study, including orphan medicines as a separate category. The total number of patients studied and the number of patients with long-term use (6 and 12 months) for chronic medication before approval were extracted. In addition, information regarding the type of Marketing Authorisation (MA), type of molecule, Anatomical Therapeutic Chemical (ATC) code and additional pharmacovigilance activities were extracted from the European Public Assessment Report (EPAR).

**Results:**

A total of 498 new medicines were identified, of those 322 were non-orphan medicines and 176 were orphan medicines. The median total number of patients studied before approval was 1,787 (interquartile range [IQR] 781–3,351) for non-orphan medicines and 333 (IQR 139–648) for orphan medicines. In the group of non-orphans, chronic medication was studied in a larger number of patients (median 2,253, IQR 1,523–3,662) than medication for intermediate (median 798, IQR 520–1,757) or short-term use (median 2,038, IQR 807–6,110). Among the 154 non-orphan medicines intended for chronic use, 83 (54%) met the patient exposure recommendations for 6-months use, and 113 (73%) met the criteria for 12-months patient exposure. Among the 101 orphan medicines intended for chronic use, only 1 (1%) met the patient exposure recommendations for 6-months use and 7 (6.9%) met the criteria for 12-months patient exposure.

**Conclusion:**

Orphan medicines and “precision” medicines tend to have less clinical data available at the time of marketing authorisation. The median of the total number of subjects studied prior to approval has been decreasing overtime. Additional pharmacovigilance activities play, in these cases, a key role in the safety monitoring and assessment of the benefit-risk profile during the medicine’s lifecycle.

## Introduction

Safety evaluation is a central component in all stages of the drug development lifecycle. Prior to the marketing authorisation of a medicine, rigorous safety monitoring and evaluations from both preclinical and clinical development phases are required. The safety profile of the product should be adequately characterized in order to obtain regulatory approval [1].

In November of 1994, the International Council for Harmonisation of Technical Requirements for Pharmaceuticals for Human Use (ICH) approved a guideline with a set of principles for the safety evaluation of drugs intended for the long-term treatment (chronic or repeated intermittent use for longer than 6 months) of non-life-threatening diseases. This E1 guideline “Note for guidance on population exposure: the extent of population exposure to assess clinical safety (CPMP/ICH/375/95)” established recommendations on three levels: total number of patients exposed should be at least 1,000, and data of 6 months of use should be available for at least 300 patients and 1 year exposure for at least 100 patients [2]. No rationale, calculations or further explanation was given in the guideline regarding the thresholds chosen for the safety assessment.

This guidance has been used since 1995 without updates. However, in the European Union (EU), the regulatory framework has changed, especially over the last two decades, to enhance innovation and ensure timely access to medicines. The advent of new regulatory tools, such as the expedited approval pathways, the new pharmacovigilance regulation implemented in 2012, the increasing number of advanced therapy medicinal products (ATMPs), and the increasing importance of real-world evidence in regulatory decisions has re-shaped the research and development of new medicines [3–5].

The aim of this study was to review the number of patients exposed to new medicines before approval in the EU, with a special focus on long-term exposure for medicines intended for chronic use. Further, we compared our results with similar research performed by Duijnhoven R. *et al*., for the period 2000–2010 [6], in order to assess if there was a difference in the total number of patients exposed between different groups of medicines.

## Methods

The publicly available search-database of the European Medicines Agency (EMA) was used to identify all products approved in the EU through the centralised procedure between 1 January 2011 and 31 December 2023, including those that were subsequently withdrawn or suspended [7]. We included all unique, new active substances that were approved in this period. Duplicate products were excluded. Duplicates were defined as all medicines with an identical active substance, and with the same dossier and the same preclinical and clinical studies, but with two or more product names. European public assessment reports (EPARs) are publicly available on the EMA’s website [8]. From the EPARs for all products, we extracted the total number of participants in the studies (patients, as well as healthy volunteers) who received at least one dose of the medicine. The intended use of medications was assessed based on the official indication at approval. With this indication as a reference, intended treatment duration was classified as chronic (when medication is meant for >1 year [9,10]), intermediate (to be used > 1 month but not chronically), or short term (medication to treat an acute condition, usually to be used less than a month [11,12]). Examples of chronic use included asthma and HIV medication, intermediate length of use included anticancer treatment, and short-term use included antimicrobial medication, and most analgesics and diagnostic agents. For all medicines intended for chronic use, we extracted additional information on the number of patients who had received treatment for at least 6 months and at least 12 months. If no information on the number of exposed patients could be obtained, patient exposure was categorised as missing. In addition, information was obtained on ‘orphan drug’ status and type of marketing authorisation (MA) granted (standard, under exceptional circumstances, and conditional approval). Products were categorised as ‘orphan medicines’ if they had an orphan drug designation (ODD) at the time of Committee for Medicinal Products for Human Use (CHMP) opinion, even if the applicant requested the removal of the ODD at the time of the MA granting. All other medicines were categorised as ‘non-orphan medicines’. From the EPARs we extracted information regarding the registration status (approved or withdrawn), the type of the initial marketing authorization (standard, conditional approval or approval under exceptional circumstances), type and subtype of molecule (small molecule, synthetic peptide, biological – vaccine, advanced therapy medicinal product, ATMP or other – oligonucleotide, radionuclide or herbal preparation) ATC code, and additional pharmacovigilance activities (category 1, 2 or 3). As defined in the guidance on format of the Risk Management Plan (RMP) in the EU(9), category 1 are imposed mandatory additional pharmacovigilance activities which are conditions of the marketing authorisation; category 2 are imposed mandatory additional pharmacovigilance activities which are specific obligations in the context of a conditional marketing authorisation or a marketing authorisation under exceptional circumstances; category 3 are required additional pharmacovigilance activities [13]. For the products withdrawn we checked the withdrawal letter, published on EMA website, to retrieve the reason for the withdrawal, as this could be related to safety issues. Based on the total number of participants exposed (healthy volunteers and patients) before approval, all products were divided into one of the following five groups: less than 500 patients, 500–999 patients, 1,000–1,999 patients, 2,000–5,000 patients, and more than 5,000 patients. To assess long-term use before approval, the numbers of participants studied for at least 6 months and for at least 12 months were calculated. The cutoff values used for the number of patients required in long-term studies were chosen according to the clinical safety guideline: at least 300 for 6-months use and at least 100 for 12-months use [2,14].

The non-parametric Mann-Whitney test was used to explore whether there was a statistically significant difference: i) in the number of participants studied for medicines still approved versus those withdrawn (for all reasons) (sign off date: 18 October 2025); ii) in the number of participants exposed for non-orphan medicines versus those with orphan drug designation; iii) in the number of participants exposed in case of small molecules versus non-small molecules; iv) in the number of participants exposed in case of standard MA’s versus non-standard MA’s (e.g., conditional marketing authorisation and marketing authorisation under exceptional circumstances); v) in the number of patients exposed to medicines with oncological indications versus patients exposed to medicines with non-oncological indications; vi) in the number of patients exposed to vaccines versus patients exposed to non-vaccine products.

We applied the non-parametric chi-square test to assess whether a total number of patients exposed prior to approval lower than 1000 was predictive of a post-authorisation commitment. The statistical analysis was performed using SPSS version 29.0 Software Program.

## Results

We identified 498 newly approved medicines in the period 2011–2023, of which 322 (65%) were standard (non-orphan) medicines and 176 (35%) were orphan medicines. The specific medicines and number of subjects studied are listed in Dataset S1 File.

### Total number of patients exposed

**Fig 1:**
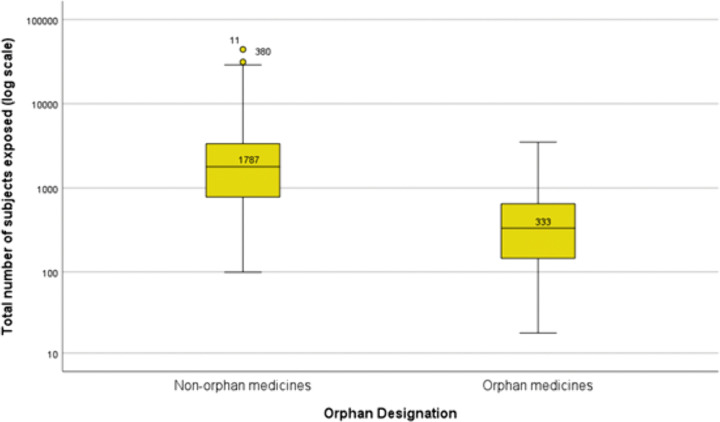
Boxplots with medians of the number of patients studied prior to approval (non-orphan medicines, n = 322 versus orphan medicines, n = 176). Boxplots present the 50^th^ percentile, i.e., the median value is given, with the interquartile range (25^th^ and 75^th^ percentiles) indicated by the box, the 2^nd^ and 98^th^ percentiles indicated by the horizontal bars of the whiskers, and outliers indicated by individual circles. The total number of patients studied (y-axis) is plotted on a logarithmic scale.

The median number of the total of patients studied per medicine was 1,787 (interquartile range [IQR] 781–3351) for non-orphan medicines (n = 322) and 333 (IQR 139–648) for orphan medicines (n = 176) (Fig 1). This difference was statistically significant (p < 0.001; Mann-Whitney test).

**Fig 2:**
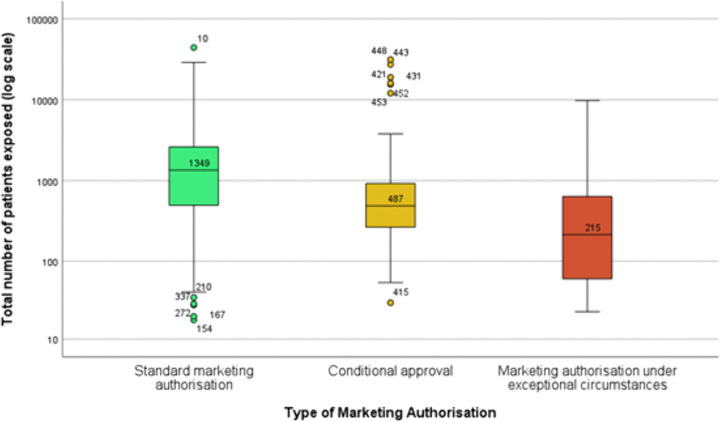
Boxplots with medians of the number of patients studied prior to approval according to the type of Marketing authorisation: standard (n = 392), conditional (n = 78) or approval under exceptional circumstances (n = 28). Boxplots present the 50^th^ percentile, i.e., the median value is given, with the interquartile range (25^th^ and 75^th^ percentiles) indicated by the box, the 2^nd^ and 98^th^ percentiles indicated by the horizontal bars of the whiskers, and outliers indicated by individual circles. The total number of patients studied (y-axis) is plotted on a logarithmic scale.

If we consider the type of MA granted, the median of the total number of patients studied per medicine was 1,349 (IQR 496–2608) for the group of medicines with a standard MA (n = 392), 487 (IQR 266–913) for medicines with a conditional approval (n = 78) and 215 (IQR 66–583) for medicines approved under exceptional circumstances (n = 28) (Fig 2). This difference was statistically significant (p < 0.001; Mann-Whitney test).

**Fig 3:**
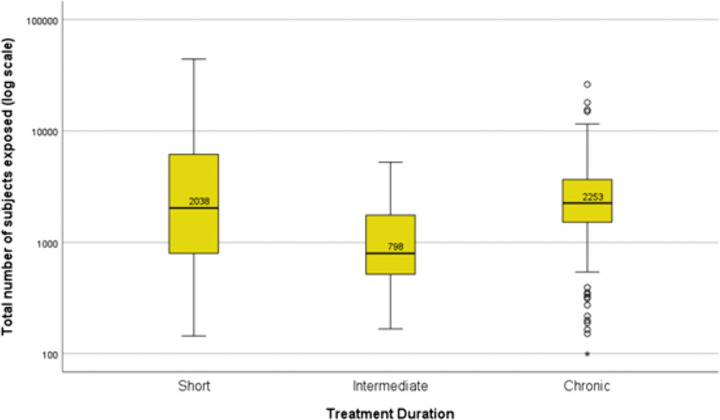
Boxplots with medians of the number of patients studied prior to approval. Results for non-orphan medicines (n = 322) are here presented by intended length of use of the products (short-term, n = 82, intermediate, n = 86, or chronic, n = 154). Boxplots present the 50^th^ percentile, i.e., the median value is given, with the interquartile range (25^th^ and 75^th^ percentiles) indicated by the box, the 2^nd^ and 98^th^ percentiles indicated by the horizontal bars of the whiskers, and outliers indicated by individual circles. The total number of patients studied (y-axis) is plotted on a logarithmic scale.

In the subgroup of non-orphan medicines (n = 322), the median number of total patients studied per medicine was 2,253 (IQR 1,523−3,662) for medicines intended to treat chronic diseases (n = 154), 798 (IQR 520−1,757) for medicines intended to be used for an intermediate period (n = 86) and 2,038 (IQR 807−6,110) for medicines used for short periods (n = 82) (Fig 3).

**Fig 4:**
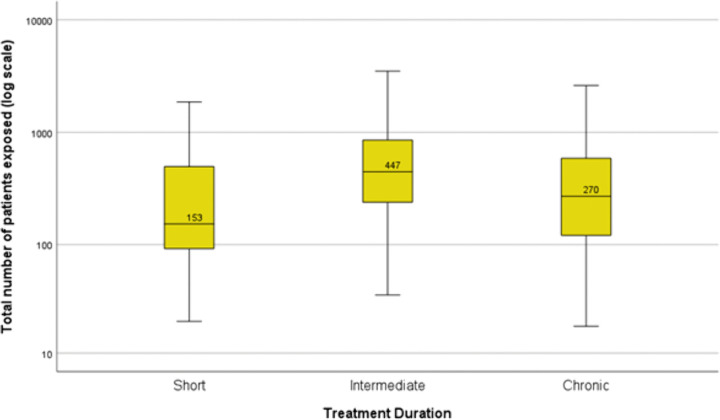
Boxplots with medians of the number of patients studied prior to approval. Results for the orphan medicines (n = 176) are here presented by intended length of use of the products (short, n = 25, intermediate, n = 50, and chronic-term, n = 101). Boxplots present the 50th percentile, i.e., the median value is given, with the interquartile range (25th and 75th percentiles) indicated by the box, the 2nd and 98th percentiles indicated by the horizontal bars of the whiskers, and outliers indicated by individual circles. The total number of patients studied (y-axis) is plotted on a logarithmic scale.

In the subgroup of orphan medicines (n = 322), the median number of total patients studied per medicine was 270 (IQR 121–588) for medicines intended to treat chronic diseases (n = 101), 447 (IQR 243–842) for medicines intended to be used for an intermediate period (n = 50) and 153 (IQR 92–497) for medicines used for short periods (n = 25) (Fig 4).

Orphan medicines generally had small numbers of patients in clinical studies; 140 of the 167 (84%) of the products had been tested by less than 1,000 patients (Table 1).

**Table 1:** Number (percent) of non-orphan and orphan medicines categorised according to total number of individuals studied prior to marketing authorisation.

Total Number of patients	Non-orphan medicines (n = 322)				Orphan Medicines (n = 176)				Total (n = 498)
	*Chronic (n = 154)*	*Intermediate (n = 86)*	*Short-term (n = 82)*	*Sub-total* *(n = 322)*	*Chronic (n = 101)*	*Intermediate (n = 50)*	*Short-term (n = 25)*	*Sub-total*	
**<500**	13 (8.4%)	19 (22.1%)	12 (14.6%)	44 (13.7%)	70 (69.3%)	28 (56%)	19 (76%)	117 (66.5%)	161 (32.3%)
**500-999**	12 (7.8%)	32 (37.2%)	14 (17.1%)	58 (18.0%)	16 (15.8%)	13 (26%)	2 (8%)	31 (17.6%)	89 (17.9%)
**1,000-1,999**	40 (26.0%)	19 (22.1%)	15 (18.3%)	74 (23.0%)	12 (11.9%)	6 (12%)	4 (16%)	22 (12.5%)	95 (19.1%)
**2,000-5,000**	68 (44.2%)	16 (18.6%)	17 (20.7%)	101 (31.3%)	3 (3.0%)	3 (6%)	0 (0.0%)	6 (3.4%)	107 (21.5%)
**>5,000**	21 (13.6%)	1 (1.2%)	24 (29.3%)	46 (14.3%)	0 (0.0%)	0 (0.0%)	0 (0.0%)	0 (0.0%)	46 (9.2%)
Total	**154 (100%)**	**86 (100%)**	**82 (100%)**	**322 (100%)**	**101 (100%)**	**50 (100%)**	**25 (100%)**	**176 (100%)**	**498 (100%)**

The number of participants receiving medicines for short-term treatment before marketing authorisation varied considerably.

Medicines intended for intermediate length of use were tested in the smallest number of patients before approval; 75 of the 90 (83.4%) medicines were used by less than 2,000 patients. And within this category, 78 of the 90 (86.7%) medicines were indicated for treatment of cancer.

Medicines for chronic use were studied in larger numbers of patients during clinical development. In total, 89 of the 154 (57.8%) of these products had been used by 2,000 or more patients, of which 21 of the 154 (13.6%) had been studied in more than 5,000 patients.

In our dataset, 245 of the 492 (49.8%) medicines identified were approved with a total number of patients exposed lower than 1,000 (Table 1). From these, 140 of the 245 (57%) are orphan medicines.

### Trends in patient exposure prior to approval (2011–2023)

**Fig 5:**
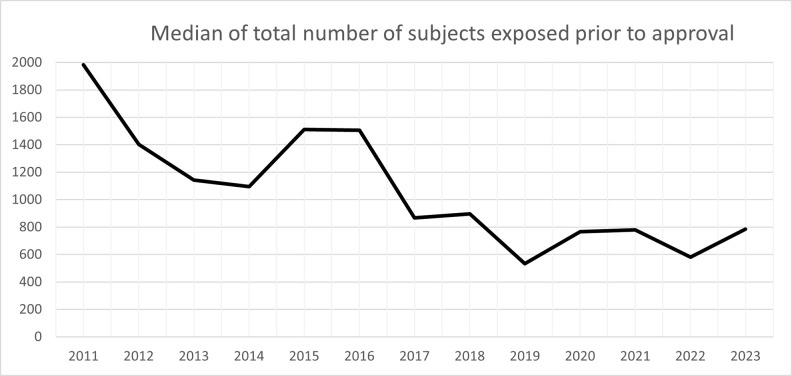
Medians of the total number of patients studied prior to approval for the period 2011-2023.

The median number of patients exposed to new active substances prior to approval shows a decreasing trend over the period 2011–2023, particularly from 2016 onwards (Fig 5). In 2011, the median number of exposed subjects was approximately 1,900, while from 2017 onwards, the median generally remained below 900 subjects.

### Difference in the median of total patients between different groups of medicines

#### Medicines approved versus withdrawn.

Over the study period, 42 of the 498 (8.4%) medicines had their marketing authorisation withdrawn. Medicines still on the market had been studied prior to approval in a median 947 patients (IQR 379–2,286) versus 1,710 patients (IQR 364–2,727) for withdrawn medicines; this difference was not statistically significant (p = 0.244; Mann-Whitney test).

#### Small molecules versus non-small molecules.

199 of the 498 (40%) medicines in our analysis are non-small molecules, i.e., biologicals (excluding vaccines, in this analysis), oligonucleotides, peptides or radionuclides. Medicines which are small molecules had been studied before approval in a median 1,279 patients (IQR 584–2,402), versus 475 patients (IQR 169–1599) for non-small molecules; this difference was statistically significant (p < 0.001; Mann-Whitney test).

#### Standard MA versus non-standard MA.

106 of the 498 (21.3%) medicines in our analysis received a non-standard marketing authorisation, i.e., conditional approval or approval under exceptional circumstances. Medicines with a standard MA had been studied prior to approval in a median 1,349 patients (IQR 496–2,608) versus 462 patients (IQR 171–862) for non-standard MA; this difference was statistically significant (p < 0.001; Mann-Whitney test).

#### Conditional MA versus MA under exceptional circumstances.

From these 106 medicines with a non-standard MA, 78 (74%) have a conditional approval and 28 (26%) have received an approval under exceptional circumstances. Medicines with a conditional approval had been studied prior to approval in a median 487 patients (IQR 266–913) versus 215 patients (IQR 66–583) for medicines approved under exceptional circumstances; this difference was statistically significant (p < 0.011; Mann Whitney test).

#### Oncological indications versus non-oncological indications.

149 of the 498 (30.0%) medicines in our analysis are intended to treat oncological diseases. Medicines with oncological indications (orphans and non-orphans) had been studied prior to approval in a median 588 patients (IQR 374–1,114), versus 1,507 patients (IQR 379–3,156) in case of medicines with non-oncological indications; this difference was statistically significant (p < 0.001; Mann-Whitney test).

#### Vaccines versus non-vaccines.

29 of the 498 (5.8%) medicines in our analysis are vaccines. Vaccines had been studied before approval in a median 9,772 patients (IQR 4,552–16,689), versus 886 patients (IQR 355–2,034) in case of non-vaccine products; this difference was statistically significant (p < 0.001; Mann-Whitney test).

### Long-term studies of medicines for chronic use (non-orphan medicines)

**Fig 6:**
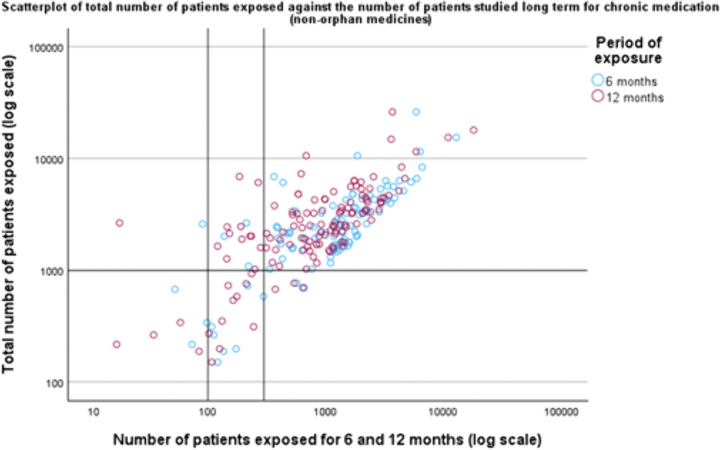
Scatterplot displaying the total number of patients studied prior to approval plotted against the number of patients studied long term (for 6 and 12 months) for chronic medication (for non-orphan medicines). Reference lines are added to indicate the minimum criteria from the ICH E1 guideline: 1,000 patients in total and 300 and 100 patients studied for 6 and 12 months, respectively.

Among the 154 non-orphan medicines intended for chronic use 83 (54%) met the patient exposure recommendations (at least 300 participants studied for 6 months and at least 1,000 participants in total), and 113 (73%) of the medicines met the criteria for 12-months patient exposure (at least 100 participants) (Table 2; Fig 6).

**Table 2:** Number (percent) of non-orphan medicines categorised according to total number of individuals studied for 6 and 12 months (long term) prior to marketing authorisation.

Total Number of patients	Number of patients with ≥ 6-months use				Number of patients with ≥ 12-months use			
	<300	300−1,000	>1,000	Missing	<100	100−1,000	>1,000	Missing
**<1,000 (n = 25)**	10 (40%)	2 (8%)	0 (0,0%)	13 (52%)	4 (16%)	13 (52%)	0 (0.0%)	8 (32%)
1,000-5,000 (n = 108)	4 (3.7%)	19 (17.6%)	47 (43.5%)	38 (35%)	1 (0.9%)	51 (47.2%)	41 (38.0%)	15 (13.9%)
>5,000 (n = 21)	0 (0.0%)	2 (9.5%)	13 (61.9%)	6 (28.6%)	0 (0.0%)	4 (19.0)	17 (81%)	0 (0.0%)
Total (n = 154)	**14 (9.1%)**	**23 (14.9%)**	**60 (39.0%)**	**57 (37.0%)**	**5 (3.2%)**	**68 (44.1%)**	**58 (37.7%)**	**23 (14.9%)**

### Long-term studies of medicines for chronic use (orphan medicines)

**Fig 7:**
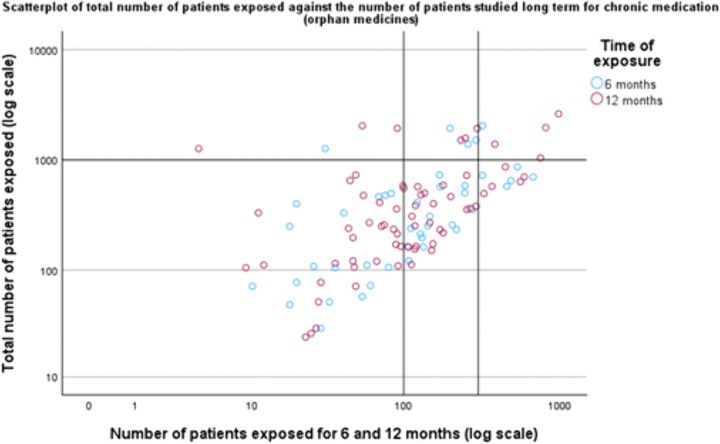
Scatterplot displaying the total number of patients studied prior to approval plotted against the number of patients studied long term (for 6 and 12 months) for chronic medication (for orphan medicines). Reference lines are added to indicate the minimum criteria from the ICH E1 guideline: 1,000 patients in total and 300 and 100 patients studied for 6 and 12 months, respectively.

Among the 101 orphan medicines intended for chronic use, only 1 (1%) met the patient exposure recommendations for 6-months use (at least 300 participants studied for 6 months and at least 1,000 participants in total) and 7 (7.2%) of the medicines met the criteria for 12-months patient exposure (at least 100 participants) (Table 3; Fig 7).

**Table 3:** Number (percent) of orphan medicines categorised according to total number of individuals studied for 6 and 12 months (long term) prior to marketing authorisation.

Total Number of patients	Number of patients with ≥ 6-months use				Number of patients with ≥ 12-months use			
	<300	300−1,000	>1,000	Missing	<100	100−1,000	>1,000	Missing
<1,000 (n = 86)	35 (40.7%)	5 (5.8%)	0 (0.0%)	46 (53.5%)	34 (39.5%)	28 (32.6%)	0 (0.0%)	24 (27.9%)
1,000–5,000 (n = 15)	5 (33.4%)	1 (6.7%)	0 (0.0%)	9 (60%)	3 (20%)	7 (46.7%)	0 (0.0%)	5 (33.4%)
>5,000 (n = 0)	0 (0.0%)	0 (0.0%)	0 (0.0%)	0 (0.0%)	0 (0.0%)	0 (0.0%)	0 (0.0%)	0 (0.0%)
Total (n = 101)	**40 (39.6%)**	**6 (5.9%)**	**0 (0.0%)**	**55 (54.5%)**	**37 (36.6%)**	**35 (34.7%)**	**0 (0.0%)**	**29 (28.7)**

### Additional pharmacovigilance activities

410 out of the 498 (82%) medicines identified have been approved with additional pharmacovigilance activities foreseen in the Risk Management Plan (RMP). Regarding the category of additional pharmacovigilance activities, in 43 (10.5%) products category 1 activities were reported, 54 (13.2%) products with category 2 and 305 (74.4%) products with category 3.

178 out of the 498 (35.7%) medicines were approved with one or more post-authorisation safety commitments, in order to collect more data and generate evidence about the safety and effectiveness of the product. Among these, 97 (52.2%) are orphan medicines and 100 (56.2%) products received non-standards MA (i.e., conditional approval or approval under exceptional circumstances). The Fisher exact test indicates that there is a statistically significant difference between the expected frequencies and the observed frequencies, with p < 0.001.

## Discussion

In our study, which included 498 medicines approved in the European Union between 2011 and 2023, the median of the total number of patients studied prior to approval was 1,787 (IQR 781–3,351) for non-orphan medicines and 333 (IQR 139–648) for orphan medicines. In the study of Duijnhoven R. *et al*. [6], which included 200 medicines approved between 2000 and 2010, the median of the total number of patients studied prior to approval was 1,708 (IQR 968–3,195) for non-orphan medicines and 438 (IQR 132–915) for orphan medicines. Overall, the results for the total number of patients exposed are similar in both studies, for both non-orphan and orphan medicines.

The ICH E1 guideline [2]sets recommendations on three levels of patient exposure for new active substances before approval: a total patient exposure of at least 1,000–1,500 patients, 6 months of use by 300 patients, and 12 months of use by 100 patients.

When the ICH E1 guideline was issued (in the 90’s) most of the medicines were small molecules. Nowadays, the pharmaceutical industry is aiming at a broad mix of different size and complexity of molecules to tackle human diseases, including small molecules, antibodies, nucleic acids, and cell and gene therapy, among others [15]. The increasing scientific knowledge and technological innovation in the areas of cell biology and biotechnology resulted in the development of tailored therapeutic approaches for the treatment of several diseases, including, serious diseases with an unmet medical need [16–18]. The ATMPs class integrates gene therapy medicinal products, somatic cell therapy medicinal products and tissue engineering products and are often individualized and patient-specific products [19]. The distinctive characteristics and features of ATMPs raised the need for long-term efficacy and safety follow up, based on prolonged biological activity and/or persistence of cells. This justified the creation of a specific EMA guideline to address quality, non-clinical and clinical requirements for investigational ATMPs (currently under public consultation) [20]. Similar to the EMA Guidelines, also in the United States a Guidance has been drafted in 2024 to address the particularities of cellular and gene therapy products [21].

The observed decline in the median number of patients exposed to new medicines prior to approval between 2011 and 2023 reflects these evolving trends in the European medicine’s development landscape. Over this period, there has been an increasing proportion of approved medicines targeting rare diseases or highly specific patient subpopulations (i.e. ATMPs), often under the orphan designation. In parallel, the EMA has expanded the use of accelerated and adaptive regulatory pathways, such as conditional marketing authorisation and approvals under exceptional circumstances [3], which may rely on smaller and shorter clinical development programmes. Advances in precision medicine and biomarker-driven drug development have also contributed to smaller trial populations, as patient selection becomes more refined. While this trend enables faster patient access to innovative therapies, it underscores the growing importance of robust post-authorisation evidence generation to complement the more limited pre-approval clinical experience.

In this context, it may be time to rediscuss the requirements for assessing medicines for long-term use for non-life-threatening diseases, the issue being the need to understand if these are still relevant in the current medicine’s development landscape. Are they the only way to obtain a clear picture on the safety profile of a new medicine or can an alternative approach be defined to gather more safety information? This discussion should involve regulators from all ICH geographic areas, industry, patients, and academia.

The recommendation to re-open this discussion on the long-term follow up requirements for chronic medication was already suggested by Duijnhoven R. *et al*. in 2013 [6]. Following the same methodology, this study shows that the percentage of medicines intended to be used chronically and for which the number of patients exposed was under the recommended threshold has been raised (from 20% to 46%). Therefore, in light of the current medicine’s development paradigm, it is emphasized the need for a new broad discussion on this topic. Biologicals (including ATMPs) have higher complexity, display a certain degree of variability (microheterogeneity) and the potential immunogenicity is considered an important safety concern [22–24]. Therefore, to follow specific guidelines for each type of molecule (small molecules, vaccines and ATMPs) seems to be more adequate. Also, the threshold of 300 patients for 6 months follow-up and 300 patients for 12 months follow-up could be discussed, since these sample sizes are not sufficient to detect long-term events. Instead, observational studies, and real-world evidence generated from RWD available could play a more prominent role as part of the dossier of the marketing authorisation for regulatory decision-making. An updated ICH E1 guideline introducing additional requirements aligned with the modern medicine’s development landscape could be of relevance: 1) the recommendation to perform post-authorisation studies when the total number of patients exposed prior to approval is lower than the threshold; 2) well-designed real-world studies to assess the long-term safety when the minimum number of patients exposed for 6 months and 12 months did not reach the threshold.

In 2005, legislation was introduced in EU to ensure marketing authorisation holders (MAH) prepare a RMP for each newly licensed product [25]. The aim of the RMP is to ensure a more proactive approach to pharmacovigilance by putting in place measures that allow for the early detection and minimisation of risks throughout a medicine’s lifecycle. This should, in theory, result in a reduction in the length of time for drug withdrawal in case a safety issue arises [26]. The RMP in the EU has been further enhanced by the introduction of new EU legislation for pharmacovigilance activities which became applicable in July 2012 [27].

In the period between 2002 and 2011 nineteen medicines were withdrawn in EU for safety reasons [26]. In the period studied in our research, analyzing only the medicines approved through the centralised procedure, 42 medicines have status “withdrawn”. It is important to highlight that, only one medicine (Ingenol mebutate) was withdrawn due to safety reasons (all the other 41 medicines withdrawn reported ‘commercial reasons’). Ingenol mebutate (Picato®), a gel approved in 2012 to treat actinic keratosis, a skin condition, was demonstrated to be associated with a higher incidence of skin cancer and was, therefore, withdrawn, in 2020 [28]. A total number of 1,165 patients were exposed to this product during the clinical development phase and the maximum time of exposure reported in the EPAR was 3 days. Due to this short exposure time, the risk of skin cancer was, of course, not identified during the clinical trials. Randomised clinical trials are the gold standard for efficacy assessment of a new intervention but they have also their limitations. One of these limitations is the lack of data for long-term safety [29,30]. For this reason, the RMP includes a strategy for obtaining information on the benefit-risk balance where safety concerns about missing information, important identified risks and important potential risks exist, which may be achieved through additional pharmacovigilance activities beyond adverse reaction reporting and signal detection and routine risk minimization measures [31,32].

In our dataset we observed that most of the medicines (83%) were approved with additional pharmacovigilance activities foreseen in their RMP. These additional pharmacovigilance activities include, for instance, post-authorisation safety studies (PASS) such as [33]: long-term follow-up of safety, pregnancy observational studies or in another subpopulations where the medicine is expected to be used. As expected, based on data from less than 1,000 subjects (independently of the orphan status and the type of MA) are more prone to safety commitments, as confirmed by our results.

Interesting to note that the group of medicines with a higher median of the total number of subjects exposed are the vaccines. Vaccines had been studied prior to approval in a median 9,772 patients versus 886 patients in case of non-vaccine products. Despite this, vaccines have been associated with a high level of distrust and hesitancy [34–38]. During the recent COVID-19 pandemic era this hesitancy and low level of trust in science, caused by misinformation, rumors and conspiracy theories, leads to some resistance to vaccination programs, inducing a decline in the intent to vaccinate [39,40]. One of the arguments for this hesitancy was that the COVID-19 vaccines received a conditional approval, arguing that the product was not “really” approved but it was still “under investigation”, using human subjects in a large-scale experimental setting [41]. Although this discussion is beyond the scope of our study research, from all categories identified in our database, vaccines were the products with the highest number of subjects exposed prior approval. This is observed even in case of vaccines with a conditional approval. Taking again as example the vaccines against COVID-19 virus infection for which a conditional approval was granted, a median of 18,904 patients were exposed prior to approval. This study endorses and re-confirms that the vaccines can be considered safe and are tested in sufficient subjects to generate robust evidence for their benefit-risk balance.

On the other hand, the group of orphan medicines, medicines intended to treat rare diseases, are, as expected, the group with the lowest median of total patients studied (333 orphans vs 1,787 in the non-orphans group) [3]. Similar results have been reported by R. Duijnhoven *et al*.

When comparing the group of medicines with oncological indications with the medicines with non-oncological indications we observe that the median of the total number of patients exposed to the medicines with oncological indications is lower than in the group of medicines with non-oncological indications. This is probably because 61 of the 148 (41%) of the medicines with oncological indications are orphan medicines. Moreover, the higher level of uncertainty associated with cancer medicines is a common reported issue raising doubts on the therapeutic value of these medicines. Different authors have investigated the level of evidence on benefit-risk profiles of cancer medicines and concluded that regulators often deemed early and less complete evidence on benefit-risk profiles of cancer drugs sufficient to grant regular approval [42]. By definition, when a new medicine receives a conditional approval, the authorisation is based on less comprehensive data than in a standard MA. Some researchers like Hoekman *et al*., concluded that the use of a conditional approval for oncology medicines deviates from *a priori* policy objectives. A conditional approval seems not being used by companies as a prospectively planned pathway, but rather to provide a way out for companies and regulators when data are not comprehensive enough to justify a full marketing authorisation [43,44].

It is also not a surprise that medicines with an approval under exceptional circumstances showed a lower median of the total number of patients exposed compared with those with a conditional MA. The conditional approval, created in 2006, is a “expedited” regulatory pathway for medicines intended to treat severely debilitating or life-threatening diseases, orphan diseases, or emergency situations [25,45]. To ensure that comprehensive data will become available in the post-authorisation phase, the MAH of a medicine approved through a conditional approval will need to fulfill “specific obligations”, and it is expected that after the submission of the results of the on-going (or new) studies the conditional approval can be converted in a standard MA. In contrast, authorisation under exceptional circumstances recognizes situations in which obtaining comprehensive data may not be possible [46].

In the group of the non-small molecules, which includes biologicals (excluding the vaccines), and oligonucleotides, we observe a lower median of total patients exposed to the drug prior to approval. In our data set, almost 50% of the non-small molecules approved have orphan designations which have contributed to the low median observed in this group. Moreover, ATMPs, for example, are generally highly individualized therapies (“precision” medicines) targeting not only rare diseases but also other high specific unmet medical needs. It has been reported in different studies that most ATMPs were approved based on small pivotal trials [47]. The number of biologicals and ATMPs in particular, has been increasing through the years [3] and in general a low number of patients is exposed during the clinical development phase raising questions related to the uncertainty of their therapeutic value at the time of the MA granting. For this reason, the European Commission (EC) may grant, in these cases, a conditional approval or an approval under exceptional circumstances. In the US, the two comparable expedited approval pathways applicable to medicines intended to address an unmet medical need are the ‘fast-track designation’ and the ‘breakthrough therapy designation’ [48]. In the European Union, for a conditional approval or approval under exceptional circumstances, there are always specific obligations (SOB) to be fulfilled by the MAH. These SOB are mentioned in Annex II of the EC decision and are binding. These may also be additional Pharmacovigilance activities and are included as well in the RMP (category 2 studies).

In summary, additional Pharmacovigilance activities are essential for the safety assessment and consequent benefit-risk evaluation through the medicine’s lifecycle, playing a key role in cases where there is a limited number of patients exposed prior to approval.

### Limitations

In some EPARs the time of exposure was reported as cumulative exposure (patient/years) instead of number of patients exposed for ≥6 months and ≥12 months. In that cases, no reliable information could be extracted. Therefore, we have classified the data as ‘missing’.

For comparison purposes, it should be mentioned that our methodology is slightly different that the methodology followed by Duijnhoven R. *et al.* [6] regarding the inclusion criteria for orphan medicines. In our study (as previously mentioned in the methods section) we have included in the orphan group, all medicines with orphan designation at the time of the CHMP opinion, even if later (at the time of the MA granting) these medicines loose or withdraw their orphan designation.

## Conclusion

The percentage of medicines approved, intended to be used chronically and for which the number of patients exposed was under the recommended threshold has risen (from 20% to 46%), compared with the previous decennium. Over the period studied, one medicine was withdrawn due to safety reasons.

Therefore, we can say that the risk management plans have been adequate, namely the additional Pharmacovigilance activities (interventional and non-interventional studies) which are essential for safety assessment and consequent benefit-risk evaluation through the medicine’s lifecycle. These additional pharmacovigilance activities play a key role in cases where a limited number of patients had been exposed prior to approval.

In this context, an update of the ICH E1 guideline deserves reflection and could be considered to incorporate additional requirements, for products where the clinical data are limited and/or the patient exposure requirements in terms of time and duration do not follow the ‘traditional’ thresholds. This would be important to ensure robust and high-quality evidence for the safety assessment of the therapies concerned.

## Supporting information

S1 File Data Set. Overview of all medicines included. List and details of all medicines included in this study.
(XLSX)
